# Antioxidant Paradox in Male Infertility: ‘A Blind Eye’ on Inflammation

**DOI:** 10.3390/antiox11010167

**Published:** 2022-01-16

**Authors:** Sulagna Dutta, Pallav Sengupta, Shubhadeep Roychoudhury, Srikumar Chakravarthi, Chee Woon Wang, Petr Slama

**Affiliations:** 1 Department of Oral Biology and Biomedical Sciences, Faculty of Dentistry, MAHSA University, SP2, Bandar Saujana Putra, Jenjarom 42610, Selangor, Malaysia; sulagna_dutta11@yahoo.com (S.D.); srikumar@mahsa.edu.my (S.C.); wang.chee@mahsa.edu.my (C.W.W.); 2 School of Medical Sciences, Bharath Institute of Higher Education and Research (BIHER), 173 Agaram Main Rd, Selaiyur, Chennai 600073, India; pallav_cu@yahoo.com; 3 Physiology Unit, Faculty of Medicine, Bioscience and Nursing, MAHSA University, SP2, Bandar Saujana Putra, Jenjarom 42610, Selangor, Malaysia; 4 Department of Life Science and Bioinformatics, Assam University, Silchar 788011, India; 5 Department of Animal Morphology, Physiology and Genetics, Faculty of AgriSciences, Mendel University in Brno, Zemedelska 1, 61300 Brno, Czech Republic

**Keywords:** antioxidants, inflammation, male infertility, oxidative stress, reductive stress

## Abstract

The pathophysiology of male infertility involves various interlinked endogenous pathways. About 50% of the cases of infertility in men are idiopathic, and oxidative stress (OS) reportedly serves as a central mechanism in impairing male fertility parameters. The endogenous antioxidant system operates to conserve the seminal redox homeostasis required for normal male reproduction. OS strikes when a generation of seminal reactive oxygen species (ROS) overwhelms endogenous antioxidant capacity. Thus, antioxidant treatment finds remarkable relevance in the case of idiopathic male infertility or subfertility. However, due to lack of proper detection of OS in male infertility, use of antioxidant(s) in some cases may be arbitrary or lead to overuse and induction of ‘reductive stress’. Moreover, inflammation is closely linked to OS and may establish a vicious loop that is capable of disruption to male reproductive tissues. The result is exaggeration of cellular damage and disruption of male reproductive tissues. Therefore, limitations of antioxidant therapy in treating male infertility are the failure in the selection of specific treatments targeting inflammation and OS simultaneously, two of the core mechanisms of male infertility. The present review aims to elucidate the antioxidant paradox in male infertility treatment, from the viewpoints of both induction of reductive stress as well as overlooking the inflammatory consequences.

## 1. Introduction

Male factor infertility has become one of the foremost global health issues and is said to account for 20% to 30% of global infertility cases [1,2]. The actual etiology of male infertility remains enigmatic, and a large majority of male infertility cases are still classified as idiopathic, where the underlying cause(s) remains elusive [3]. 

Significant research has supported the notion that oxidative stress (OS) serves as the core mechanism in the majority of the idiopathic male infertility cases [4,5]. Exceedingly high concentrations of reactive oxygen species (ROS) can outweigh endogenous antioxidant capacity, reaching pathological levels and interfering with male reproductive processes. While excessive ROS levels adversely affect male fertility, a physiological concentration of ROS is vital to mediate the normal sperm functions [6]. 

Numerous factors can attribute to OS-mediated male infertility, but the underlying pathways for most of the factors remain elusive [7]. These include environmental, occupational, dietary, recreational as well as habitual components, each varying in intensities and duration of exposure [8,9,10]. Smoking is a known risk factor of male infertility. Smoking causes leukocytospermia, one of the prime sources of ROS [11]. Moreover, ROS in tobacco smoke can overwhelm endogenous antioxidant defenses. Elevated seminal ROS levels in smokers renders spermatozoa more vulnerable to OS-mediated damages, compromising sperm function and ultimately male fertility [12,13]. Studies show that impacts of alcohol consumption on male reproductive functions vary depending on the amount consumed [14,15]. However, a level of alcohol beyond which the risk of male infertility increases has yet to be established [16]. Illicit drugs that impair male fertility include cocaine, marijuana, opiates (narcotics), anabolic–androgenic steroids (AAS), and methamphetamines [17,18]. Other endogenous factors, including metabolic syndromes (such as obesity and insulin resistance), psychological stress, and dietary factors also have been widely documented as causatives of male infertility, impairing the testicular redox balance, affecting the endocrine axes that regulate male reproduction, testicular histology, leading to disrupted testicular functions, sperm membrane lipid peroxidation (LPO), sperm DNA fragmentation, genetic and epigenetic modifications of sperm, sperm dysfunctions and overall deteriorated semen quality [16,19,20,21,22].

Treatment with antioxidants is the first line of defense against OS-mediated male infertility [23,24,25]. Agarwal et al. (2010) had clearly described the effects of various antioxidants on male fertility, fertilization, and pregnancy rates [25]. The authors have indicated that although several antioxidants reportedly have positive impact upon fertility parameters, further randomized controlled trials are needed to demonstrate the efficacy and safety of antioxidant supplements in treating idiopathic male infertility, as well as to find the optimal dose of each antioxidant to improve fertility parameters [25]. Misuse and abuse of antioxidants, “over-the-counter” usage of antioxidant supplements, and even pauciness in antioxidant efficacy are all thought to have adverse implications by transitioning the endogenous redox status to the reductive side of the scale [26]. As a consequence, any physiological extremities of the redox spectra, OS, or reductive stress, are all detrimental to male reproductive health. The term ‘antioxidant paradox’ was coined by Halliwell in the year 2000 to describe the unexpected effects of antioxidants [27]. There is little scientific literature that has put forth the explanation of ‘antioxidant paradox’ based on the importance of maintaining a delicate cellular redox balance to support physiological levels of ROS production for robust male reproductive functions [28,29]. Henkel et al. (2018) had vividly explained how the over-usage of antioxidants may cause reductive stress, and manifest contradicting impacts of antioxidant treatment on male reproduction [30]. However, there are other mechanisms that contribute to ‘antioxidant paradox’, for which in-depth explanation in the context of male infertility is needed. This article is unique in its approach to explain the vicious cycle of OS and inflammation in addressing the often failure of antioxidant treatment for male infertility. The present article supports the fact that ‘antioxidant paradox’ in male infertility treatment may partially be explained by: (i) the failure to choose treatments that specifically target both inflammation and OS; (ii) the failure to utilize both antioxidants and anti-inflammatory drugs at the same time; and (iii) the use of nonselective agents downregulating some oxidative and/or inflammatory pathways while exaggerating others.

OS is triggered by the release of reactive oxygen and nitrogen species (ROS and RNS) by inflammatory cells (which are among the major sources of ROS/RNS) at the location of the inflammation [31]. Interestingly, a variety of ROS/RNS have been reported to activate an intracellular signaling cascade that elevates the pro-inflammatory gene expressions [19,32,33]. As a result, inflammation and OS are pathophysiological phenomena that are strongly related to one another and are intimately intertwined. Failures of antioxidant treatment for male infertility roots from the failure in selection of treatment components is specific in targeting inflammation and OS, both at the same time [34]. This notion prompted us to consider the fundamental concepts of the interaction and interdependence of OS and inflammation in disrupting male reproductive functions, to explain the mechanism pertaining to ‘antioxidant paradox’ in male infertility.

## 2. Reactive Oxygen Species and Sperm Functions

OS represents a core mechanism causing male infertility, owing to its adverse impact on spermatogenesis, sperm maturation, and capacitation [6]. Followed by the endocrine orchestrated process of spermatogenesis in the testes, the produced spermatozoa is subjected to maturation in the epididymis, which is also crucial for sperm to attain fertilization-capacity and motility [35,36]. During this period, the maturing spermatozoa get exposed to endogenous ROS, which mediate various physiological functions, such as sperm hyperactivation, capacitation, and acrosome reactions [37]. These processes are crucial for proper fertilization, and they need immense energy that is derived from cellular metabolism, such as glycolysis or oxidative phosphorylation. Sperm capacitation occurs by a series of numerous cellular reactions allowing the fusion of sperm with zona pellucida of oocyte. This causes the acrosome reaction, which releases proteolytic enzymes [37].

While ROS are required for physiological sperm functions, excess ROS concentrations can overwhelm the antioxidant capacity. In the seminal fluid, the antioxidants, both enzymatic and non-enzymatic, resist the induction of OS and protect sperm from oxidative damage. Notable functions are played by the enzyme triad of superoxide dismutase (SOD), catalase, and glutathione peroxidase (GP_X_) [38,39]. SOD is a metalloenzyme that catalyzes superoxide anion dismutation processes and protects polyunsaturated fatty acids (PUFA) and sperm DNA integration [40,41,42,43]. Catalase converts hydrogen peroxide to molecular oxygen and water. Catalase possesses a heme structure with a central iron atom. Its enzymatic activities are observed in the cytoplasm, mitochondria, peroxisomes, as well as in endoplasmic reticulum. The sperm antioxidant system also includes GP_X_, whose active site contains selenocysteine. This enzyme reduces hydrogen peroxide (H_2_O_2_) [41]. It has three isoforms: nuclear, mitochondrial, and cytosolic. Sperm functions are mainly aided by the mitochondrial isoform [41,44]. If the endogenous antioxidants are unable to protect cells from high ROS, cell death can occur. Thus, ROS have a dual role: they aid sperm functions, but when in excess, they also impair sperm morphology, functions, and viability [6,45].

Sperm membranes contain a high concentration of PUFA, which ensure the fluidity required for fertilization. However, PUFA is susceptible to LPO, this high level of PUFA poses a risk of oxidative damage to spermatozoa [46]. The oxidative damage that can occur is linked to membrane fluidity loss, morphological changes in the spermatozoa, mitochondrial dysfunctions, decreased vitality, and other changes that lead to fertilization failure [46]. In mature spermatozoa, the haploid and densely compressed nucleus no longer transcribes, therefore they cannot respond to stress. During maturation phases, spermatozoa lose much of their cytoplasm, the main antioxidants source. So, spermatozoa have relatively little cytoplasm, which is mostly filled with DNA which becomes vulnerable to oxidative damage causing sperm DNA fragmentation [47].

## 3. Antioxidants in Oxidative Stress-Induced Male Infertility

Most human diseases, including ageing, cancer, and even reproductive abnormalities, include OS as a primary central mechanism. Antioxidant therapy has recently sparked substantial research interest, owing to a rapidly ageing population. Antioxidants appeal to people because they are considered ‘natural’ and thus ‘good’ chemicals, and they are connected to a healthy diet [48]. This common belief is backed up by aggressive antioxidant product marketing campaigns in a multibillion-dollar industry. Reducing OS is thought to be a method to avoid various diseases [49,50]. Given that these components can be purchased over the counter without a prescription and that antioxidant products are used to supplement diets, it is critical that they have no negative side effects. While early research on antioxidant supplements showed that they are efficient in preventing ailments, in more concurrent studies, efficacy of such therapies has been questioned. The US Food and Drug Administration (FDA) has made no recommendation for the antioxidant supplementation for any physiological illness. Furthermore, the European Society for Human Reproduction and Embryology (ESHRE) has issued a current consensus recommendation stating the insufficiency of evidence that supports the usage of antioxidant supplements [51]. Excessive supplementation has been found to be deleterious to health in various studies [52,53,54] and these concerns were supported in later studies [55]. Aside from that, it has been demonstrated that overly high quantities of antioxidants are teratogenic to embryos [56]. On the contrary, there has been research demonstrating that antioxidant therapy can help to improve male reproductive health [5,32,57,58,59,60,61]. In the current state of reproductive medicine research, antioxidant treatment for male infertility has received considerable attention (Table 1).

Oxygen metabolism leads to ROS generation, such as the superoxide anion (O_2_^●−^), H_2_O_2_, and the hydroxyl (^●^OH) radical, which bear unpaired electrons in their outer orbits, rendering these molecules unstable and highly reactive in nature [78]. In the aerobic metabolic process, oxidative phosphorylation in mitochondria leads to ROS generation, 1–5% of which seepages out and released as metabolic cytotoxic by-products [6,37,79,80,81,82,83]. As a consequence of their immense reactivity and brief half-life periods (in the nanosecond range), ROS is capable of massive disruption of biological components, including lipids, proteins, and nucleic acids [84]. The overproduction of ROS thus disrupts cellular functions and leads to a variety of pathologies, including reproductive disorders [85,86,87]. Besides the adverse impacts of ROS on cellular processes when produced in excess, they are also essential for cell growth and maturation, intracellular signaling cascades, immune responses to infectious agents and inflammatory reactions, and pertaining to male reproductive functions, ROS aid sperm maturation, capacitation, hyperactivation, acrosome reaction as well as embryonic morphogenesis [37,40,88,89,90]. Superoxide scavenging by SOD catalysis, on the other hand, prevented capacitation-related tyrosine phosphorylation in human sperm, effectively rendering them infertile [91,92]. To scavenge excessive ROS, cells generally employ a variety of intrinsic and extrinsic antioxidant mechanisms. Endogenous antioxidants comprise enzymes, such as SOD, catalase, and thiol peroxidases, besides nonenzymatic antioxidants, such as glutathione. Micronutrients include L-carnitine, coenzyme Q10 (CoQ10), vitamins A, C, E, and trace elements, such as zinc and selenium [84]. Exogenous antioxidants, on the other hand, are those that must be provided to the body exogenously to maintain a healthy redox equilibrium in every live cell [93]. Obesity, which is induced by a poor lifestyle, can lead to systemic inflammation, increased inflammatory cytokine production, and OS [94,95,96,97]. Furthermore, consuming alcohol, smoking, being exposed to radiation, and being subjected to occupational or environmental pollutants all contribute to OS [98,99].

## 4. Induction of Reductive Stress

Uncontrolled, high antioxidant exposure disrupts cellular redox balance, resulting in ‘reductive stress’. The term ‘reductive stress’ refers to a redox balance change in the body’s reductive end of redox scale [100], a circumstance that has been compared to OS in terms of its deleterious effects [101]. A recent article has explained the redox regulation in male reproduction, analyzing the impact of both the OS and reductive stress on sperm function, and explained the “antioxidant paradox” from the viewpoint of induction of reductive stress [102]. The oxidation reduction potential (ORP) is a measure of oxidizing or reducing ability of any substance. In a pioneering study, Selvam et al. had established the physiological range of ORP (−9.76 to 1.48 mV/10^6^ sperm/mL) based on the impacts of both OS and reductive stress on sperm functions and its molecular alterations [103].

**Figure 1:**
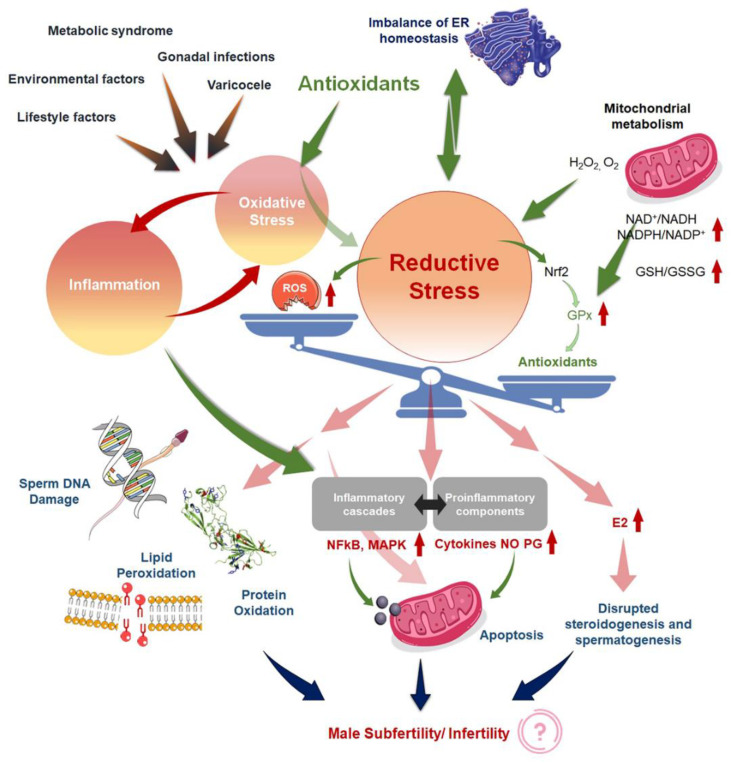
Mechanisms to explain ‘antioxidant paradox’ pertaining to male infertility, both by the induction of reductive stress and the failure to address the interconnected link of oxidative stress (OS) with inflammation. Various endogenous and exogenous factors may induce OS. OS and inflammatory pathways operate in loops, each triggering the other. Antioxidants act to mitigate excess reactive oxygen species (ROS) to minimize the adverse impacts of OS on male reproductive tissues. If overused, antioxidants may shift the redox scale towards the reductive end causing reductive stress rendering insufficient ROS needed for normal physiological functions of the sperms. Moreover, reductive stress also may revert to OS conditions. Finally, the antioxidants fail to curb the inflammatory responses that may again lead to OS and OS-induced male reproductive disruptions. ER = endoplasmic reticulum; MetS = metabolic syndrome; GSH = reduced glutathione; GSSG = oxidized glutathione; ROS = reactive oxygen species; RNS = reactive nitrogen species; GPx = glutathione peroxidase; Nrf2 = nuclear factor-erythroid factor-2-related factor-2; E2 = estradiol; NFkB = nuclear factor kappa B; MAPK = mitogen activating factor kinase; NO = nitric oxide; PG = prostaglandin.

In the case of sufficient reducing equivalents, an increase in the NAD^+^/NADH (oxidized and reduced forms of nicotinamide adenine dinucleotide) and/or GSH/GSSG (reduced and oxidized forms of glutathione) ratio or antioxidant enzyme overexpression can eliminate all ROS, resulting in an H_2_O_2_ surplus and leakage from mitochondria, urging the cells to reductive stress [104] (Figure 1). Excess NADH can disrupt mitochondrial homeostasis, cause pathological mitochondrial oxidation, and misfolding of protein in the endoplasmic reticulum [105]. Proper redox balance is essential to mediate the perfect levels of induction and restrictions to ascertain appropriate protein folding as well as the creation of disulfide bonds, that are important to determine normal structural and functional aspects of proteins [106]. The ‘unfolded protein response of the endoplasmic reticulum’ (UPR^ER^) is triggered when reductive stress impairs disulfide bond formation [107]. Proteostasis in this compartment must be recovered by restoring correct protein folding [107]. Chronic reductive stress can potentially lead to OS, which then drives reductive stress through a feedback loop. When electron acceptors are anticipated to be greatly reduced during reductive stress, various redox proteins can transfer electrons to O_2_, enhancing ROS generation [108]. As a result, this is a two-edged sword. Excessive reducing equivalents stifle cellular growth, limit mitochondrial activity, alter protein disulfide bond formation, and impede cellular metabolism [26]. Reductive stress has previously been acknowledged in other medical domains to explain pathological conditions, such as carcinogenesis, cardiomyopathy, cerebral microvasculature, blood-brain barrier dysfunction, and neurodegenerative diseases [109,110,111], and the same may also apply for male infertility.

## 5. Antioxidants-Induced Reductive Stress

As previously discussed, antioxidant supplementation is one of the most common treatment strategies to address OS-related diseases, including male infertility. Antioxidants, in contrast to oxidants, which are electron acceptors, are chemicals that inhibit oxidation by donating electrons [112]. To sustain homeostasis, the redox potential of the body must be balanced. The equilibrium of ROS, free radical generation, and antioxidant levels must be maintained. To do this, the human body must consume antioxidants through diet, or antioxidant supplements should be used if this is insufficient due to oxidant exposure. Thus, dietary antioxidants must be distinguished from antioxidant supplements, which are well-publicized and easily available over-the-counter. Patients commonly ingest excessive amounts of over-the-counter antioxidants, which are frequently touted as health boosters because they may ‘combat’ pathological illnesses. Exogenous supplements typically contain large amounts of a few refined antioxidants, for instance, vitamins A, C, E, and lycopene. Furthermore, many common foods already contain a variety of vitamins and antioxidants. As a result, it is conceivable that patients will inadvertently take very high doses of antioxidants, or even excessively high doses [23]. Supplementing with antioxidants, on the other hand, has not always yielded favourable outcomes. Furthermore, several studies have shown that antioxidant consumption has detrimental implications. Vitamin E, for instance, has been reported to elevate all-cause mortality in people who take higher doses [113]. Furthermore, it is widely identified that vitamin A supplement has no anticancer effect, whereas in smokers contradictory results are reported [114]. Vitamin C deficiency have been reported to lead to increased OS and DNA fragmentation [115], while excess of vitamin C, on the other hand, may also induce OS [116]. In summary, these contradictory findings focus the significance of ROS production and antioxidant scavenging for optimal physiological processes, that include regulation of male reproduction. As a result, a delicate cellular redox balance is essential to sustain this ‘homeostasis’ [28,29].

Studies have demonstrated a considerably good impact of additional intakes of antioxidants, including vitamins C, E, L-carnitine, and/or CoQ10 on sperm DNA integrity and semen parameters [4,117,118,119,120]. Supplementing infertile men with antioxidants may advance pregnancy outcomes and reduce live birth rates, according to a study by Showell et al. [121]. Huang and colleagues discovered that seminal OS, which is caused by low antioxidant levels, is associated with male infertility. Antioxidant therapy is proven to be more efficient in the management of idiopathic and varicocele-mediated male infertility [122,123]. Although it is clear that antioxidants are necessary for the prevention of OS, some experts believe that such interventions have little or even adverse impact on sperm maturation and the normal physiological functions of sperm, such as sperm motility, capacitation, and acrosome reactions, which lead to deteriorated semen quality [74,124]. While ROS play an important role in the etiology of several human diseases, larger doses of antioxidant supplements have resulted in paradoxical results, prompting scientists to create the term ‘antioxidant paradox’ [27]. Once again, this highlights the gap in knowledge about the mechanisms by which antioxidant treatments work.

Since key sperm functions, such as capacitation and acrosome reaction, require a physiological amount of ROS, maintaining the redox balance is important for sperm. Excess antioxidants will quench these effects, preventing sperm from fertilizing oocytes [28,29]. In this regard, elevated antioxidant levels have been associated with the development of mammalian embryos [56,125]. Because these early embryos are generally exposed to an hypoxic environment in the womb, glycolytic energy generation appears to be beneficial for cellular compaction and blastulation [126]. The metabolic shifts may cause variations in the expressions of redox-sensitive transcription factors and genes. Critical embryonic development processes starting from fertilization, genome activation, and cellular differentiation, may be disrupted as a result of this. Reproductive redox biology is still a poorly understood area. Menezo and colleagues investigated whether daily supplementation with a combination of vitamins A, C, and E, selenium, and zinc enhanced sperm DNA damage [69]. Sperm nuclear decondensation, on the other hand, increased, most likely as a result of vitamin C-induced disulphide bonds decrease in protamines [127]. As a result, the chromatin becomes destabilized, resulting in failed fertilization.

As previously mentioned, several of the commonly recommended antioxidants, for instance selenium and vitamins C and E, have been linked to negative health outcomes [128]. There is yet to be any evidence of a selenium deficiency in the scholarly literature. Higher levels of seminal plasma selenium (80 ng/mL) are linked to reduced sperm motility, asthenozoospermia, and high rates of abortion in some studies, whereas 40 and 70 ng/mL of selenium have been found to be ideal for reproductive efficiency (e.g., higher pregnancy outcomes and lower rates of abortion) [129]. In fact, there is mounting indication that the antioxidant or pro-oxidant activities are ultimately dependent on their concentration, even when they are derived from natural sources [130]. Due to the synergistic activity of many herbal antioxidant compounds, when the synergistic molecule is missing, antioxidant therapy may turn from ineffective to highly deleterious [114,131].

Menezo et al. reported that antioxidants, particularly vitamins E and C (400 mg each), zinc (500 µmol), selenium (1 µmol), and carotene (18 mg) when given orally on a daily basis to male partners of couples who could not conceive even after IVF/ICSI (in vitro fertilization/intracytoplasmic sperm injection), showed reduced sperm DNA damage while showing increasing sperm DNA decondensation, potentially leading to asynchronous chromosomal condensation [69]. Capability of vitamin C to disrupt disulphide bonds in sperm DNA protamines may be generating more DNA decondensation in IVF and ICSI patients, resulting in poor pregnancy outcomes [127,132]. Reduced protamine levels will eventually cause nuclear decondensation problems and reduce fertility [69].

In a previous in vitro investigation, vitamin C was found to have both positive and negative dose-related effects on sperm membrane LPO and motility. Vitamin C given at concentrations below 1000 µM, showed improved motility in sperm and decreased LPO, but doses above 1000 µM had adverse effects and resulted in total sperm immobility when given at concentrations over 4000 µM [133]. These studies show that antioxidants have inconsistent effects on male infertility therapy, that is, the effect is dose-dependent [63,65,66,68,69,72,73,77,134]. Moreover, to appropriately address clinical issues of each patient, practitioners would need to be clear about their specific cellular redox status. The problems are (a) lack of established method for testing an individual’s seminal redox state, (b) lack of knowledge about the normal redox level, and (c) as a result, there are no generally agreed cutoff values for the same [38,121]. For these reasons, patients are used to consuming antioxidants for the fact that they have been convinced that antioxidants work wonders as antiaging agents and as general health improvers, and moreover, they are also being treated for male infertility by clinicians because OS is thought to be detrimental in this case.

## 6. Oxidative Stress and Inflammation: Interdependence and Overlaps

Response of the body to any form of tissue damage and injury is inflammation. This process is marked by leukocytes and other immune cells getting transported via circulation to the site of injury or infection in tissues. Enhanced circulation to the inflamed area, augmented vascular permeability (which permits larger serum substances to penetrate the tissues), and elicited leukocyte infiltration are three key alterations that occur when there is acute inflammation. The failure to eliminate the pathogens causes chronic inflammation, which is characterized by the recruitment and activation of various immune cells, such as the lymphocytes, macrophages, and others, resulting in a synchronized cytokine response. Contrary to acute inflammation, which occurs when tissue regeneration or repair occurs after the irritant is removed, chronic inflammation occurs when inflammation and healing happen at the same time rather than sequentially. Repair is usually a component of chronic inflammation because chronic inflammation is linked to irritants that degrade tissue architecture. Granulation tissue, which is made up of macrophages, fibroblasts, and new blood vessels, is commonly utilized to heal wounds [135].

One of the most pressing issues in modern medicine is the evident link between acute or chronic inflammation and the onset of infertility. With the progression of inflammatory process, there is impaired accessory gland function, sperm transport obstruction, and spermatogenesis dysregulation, all of which may lead to poor semen quality [136,137]. Proinflammatory cytokines operate locally because they are produced by locally activated cells or produced momentarily after an immune stimulus has been initiated [138]. In male gonads, cytokines are also produced physiologically and have been associated with normal functioning of the reproductive tissues [139,140], and thus, are regarded as natural seminal plasma components [141]. Testicular macrophages are the most important source of cytokines in the testes; however, it has been reported that Leydig cells and Sertoli cells also are involved in cytokines generation (interleukins 1 and 6: IL-1 and IL-6) in the male gonad [142]. The migration of leukocytes into tissues after tissue injury is regulated by cytokines, most notably tumour necrosis factor (TNF-α) [143]. TNF-α is produced primarily by macrophages and other mononuclear phagocytes and plays a function in the inflammation and activation of other leukocytes. TNF-α is a pro-inflammatory cytokine that has been linked to the activation of other leukocytes. By generating adhesion molecules and chemokines on the endothelium, TNF-α, for example, promotes the phagocytes’ microbial systems. TNF-α also causes apoptosis in cells, which is a cell death. IL-1, like TNF-α, is a critical inflammatory cytokine with functions that are comparable to those of TNF-α [144].

Inflammatory stimuli to the male genital tract results in an increase in the production of ROS. Furthermore, pathogenic bacterial strains that colonize the reproductive tract may cause an increase in the free radical generation, which may result in an inflammatory reaction in the host [145,146,147]. As free radicals are very reactive compounds with one or more unpaired electrons, they bear the ability to oxidatively damage the biomolecules that they come in contact with. When they react almost rapidly with any chemical in their environment, they trigger a series of reactions that promotes uncontrolled cellular damage [85,148].

Excess ROS must be neutralized on a continual basis to maintain normal cellular activity. When free radicals accumulate in excess, they overwhelm the antioxidant defenses of the male reproductive tract, resulting in damage to the male reproductive system [45,149]. Excess ROS are scavenged by the endogenous antioxidant system under physiological conditions, which helps to maintain the proper redox balance in the body [25]. Antioxidants produced by the body can be either enzymatic, such as catalase (CAT), SOD, or thiol peroxidase, or they can be non-enzymatic, such as glutathione [93]. When the levels of seminal plasma ROS overwhelms the endogenous antioxidant capacity, semen parameters may get adversely affected [25,30,38].

Glutathione peroxidase (GPX) and glutathione S-transferase (GST) are antioxidant genes that are essential for normal spermatogenesis and sperm functions. Other antioxidant genes include *keap1* (Kelch-like ECH associated protein 1), *Nrf2* (nuclear factor erythroid 2 related factor 2), and *SOD* [33,150,151]. In humans, functional polymorphisms in the antioxidant genes *NRF2*, *GST*, *SOD*, *CAT*, and *GP_X_* have been linked to male infertility [150].

Reduced sperm concentration, motility, and functions have been linked to seminal OS in several studies [6,152,153,154,155]. It has been discovered that cytokines, which are oxidative damage mediators, may also have an effect on the quality of sperm and male fertility at the same time. For example, the concentration of IL-12 is associated with reduced sperm density and motility [156,157]. In an earlier study conducted by the same researchers, it was discovered that the levels of IL-6 are greater in infertile men. Increased levels of certain cytokines in semen are typically related with a decrease in the overall quality of sperm [158].

## 7. ‘Antioxidant Paradox’ in the Light of the Interdependent Nature of Oxidative Stress and Inflammation

Antioxidants have failed to provide beneficial effects in human diseases that have been linked to OS for unknown reasons; however, several hypotheses have been advanced [27,159,160]. One possible explanation is that the association between OS and various human diseases is most likely not causal in many, if not all, cases, and as a result, antioxidants are rendered ineffective [159,160]. This argument is perplexing because there is a large body of literature demonstrating that OS plays a role in diseases as well as in male infertility [45,159]. According to another explanation, the type and dose of antioxidants applied in clinical trials may not have been specific enough to alleviate OS in a tissue or cell-specific manner (that is to say ‘on target’), and as a result, they either showed no effect or had harmful impacts [159,160]. As a result of the complexity and interdependence of the antioxidant network, this reasoning seems to be a valid explanation of ‘antioxidant paradox’. Examples include the antioxidant enzyme SOD, which can catalyze O_2_^●−^ but also generate various other free radicals as byproducts [39]. Vitamin E acts upon the cell membrane, whereas vitamin C works both extracellularly and intracellularly in aqueous media. Vitamin C, when consumed in higher or lesser mounts than the standard daily recommendation, are associated with free radical damage to DNA [161,162,163]. Moreover, the inability of antioxidants to produce beneficial health effects may be because antioxidants can produce harmful effects in some situations, such as in established cancer, where a sufficient amount of ROS is required to induce apoptosis of malignant cells [164]. According to a third explanation, the lack of an appropriate method of quantifying redox status has resulted in many clinical trials being inconclusive, which does not imply that antioxidants are ineffective [106]. Although the method of quantifying redox status in humans is far from perfect, many clinical trials have found that the redox status was not measured prior to the start of and after the completion of antioxidant therapy [27,160]. It should be noted that measuring one or several pro- and antioxidant markers may not provide a comprehensive measure of the redox status of the target organ or tissue, and that the systemic redox status may not accurately reflect the status of the target tissue.

A new explanation for the antioxidant paradox may be proposed in light of the interdependent nature of OS and inflammation discussed in this review. The failures of antioxidant clinical trials may be attributed to the selection of antioxidants that do not simultaneously inhibit both OS and inflammation, or to the use of nonselective agents that block some oxidative and/or inflammatory pathways while exacerbating the effects of others. It would be necessary to quantify both the redox and the inflammatory status before, during, and after the antioxidant therapy in order to establish the validity of this explanation. However, a recent study by Djuric et al. [165] has provided experimental evidence as to why a nonselective antioxidant that acts by inhibiting ROS generation and thereby nuclear factor kappa B (NF-κB) activation may fail. In a psychosocial stress-induced atherosclerotic animal model, selective targeting of NF-κB subunit cRel while maintaining the activity of the p50/p65 subunits provided an antiatherosclerotic effect by limiting the proinflammatory effect of NF-κB without impairing its anti-inflammatory and antioxidant functions [126]. Due to this finding, a nonselective antioxidant or anti-inflammatory agent that inhibits all the subunits of NF-κB are unlikely to be effective in treating the disease, and a selective inhibitor is required to be effective. Furthermore, this finding lends support to the idea that the interdependence between OS and inflammation, at least in some cases, may be a reasonable explanation for the antioxidant paradox, which has been previously proposed.

## 8. Conclusions

Seminal ROS is important to mediate normal sperm functions. However, when their concentrations overwhelm the endogenous antioxidant capacity, OS is induced causing cellular oxidative damage. Thus, antioxidant treatment finds significant relevance in the case of idiopathic male infertility or subfertility. However, often owing to inaccurate OS detection in male infertility cases, antioxidant treatment becomes arbitrary and leads to antioxidant overuse. This may induce ‘reductive stress’ that depletes ROS levels taking them below physiological levels critical for normal sperm functions. Moreover, reductive stress may also induce higher ROS generation, in turn causing OS. This phenomenon may partially explain the ‘antioxidant paradox’. Moreover, inflammation and OS are closely linked pathologies and establish a vicious loop impairing male fertility. These raise questions regarding antioxidants therapy in treating male infertility and highlights another mechanism to address ‘antioxidant paradox’. Failure in choosing treatment targeting inflammation and OS both simultaneously may be one of the main limitations in treating OS-induced male infertility. Research on combination therapies, including both antioxidant and anti-inflammatory agents simultaneously mitigating both OS and inflammatory pathways, may lead to an effective cure for OS-mediated male infertility.

## Figures and Tables

**Table 1:** Individual or combination antioxidant treatments with beneficial or no significant effects on semen quality.

Antioxidant Regime	Study Population	Sperm Parameters	Study
Zinc sulphate (220 mg) for 4 months	oligospermic males (*n* = 14)	21.4% (3/14) of patients achieved pregnancy	[62]
600 mg of vitamin E daily for 3 months	Infertility with high ROS	No change in SC, S_mot_ and S_morph_	[63]
Zinc sulphate (500 mg) for 3 months	Asthenozoospermia (*n* = 100)	Improved pregnancy (22.5%) vs. placebo (4.3%)	[64]
1 g vitamin C and 800 mg vitamin E daily for 56 days	Asthenozoospermia	No change in SC, S_mot_ and S_morph_	[65]
1 g of vitamins C and E daily for 2 months	Idiopathic infertility (38 infertile men with previous IVF/ICSI)	No change in SC, S_mot_ and S_morph_	[66]
L-carnitine 3 g daily; L-acetyl carnitine 3 g daily; L-carnitine (2 g daily) and L-acetyl carnitine combination (1 g daily)	Idiopathic asthenozoospermia (Placebo group = 15, L-carnitine group = 15; L-acetylcarnitine group = 15; L-carnitine and L-acetylcarnitine combined group = 15)	Increase in sperm motility and normal sperm morphology	[67]
1 g Carnitine and 500 mg L-acetyl carnitine daily for 24 weeks	Asthenozoospermia	No improvement in S_mot_	[68]
400 mg each vitamins C and E, 18 mg β-carotene, 500 μmol zinc, 1 μmol selenium daily for 90 days	38 infertile men with At least two failed IVF or ICSI	Increased chromatin decondensation	[69]
Menevit (lycopene, vitamins E, C, zinc, selenium, folate, garlic oil) daily for three months	60 infertile men	No significant difference in DFI	[70]
300 mg selenium daily for 48 weeks	Normozoospermia	No improvement in S_mot_ and S_morph_	[71]
Menevit (lycopene, vitamins E, C, zinc, selenium, folate, garlic oil) daily for three months	50 infertile men with elevated OS	No change in SC, S_mot_ and S_morph_	[72]
500 mg L-carnitine, 60 mg vitamin C, 20 mg coenzyme Q10, 10 mg vitamin E, 200 µg vitamin B, 91 µg vitamin B12, 10 mg zinc, 50 µg selenium once daily for 3 months	Prospective observational study with 20 men with grade 1 varicocele and primary or secondary infertility	No change in SC, S_mot_ and S_morph_	[73]
30 mg vitamin C, 5 mg vitamin E, 0.5 µg vitamin B12, 750 mg L-carnitine, 10 mg coenzyme Q10, 100 µg folic acid, 5 mg zinc, 25 µg selenium twice daily for 6 months	7 infertile men with sperm DFI >25%Treatment group (37 patients) Placebo group (40 patients)	No change in SC, DFI	[74]
Coenzyme Q10 = 200 mg daily	Idiopathic infertility (Placebo group = 114, Treatment group = 114)	Increase in sperm concentration, motility, and normal sperm morphology	[75]
L-carnitine = 1 g, L-acetylcarnitine = 0.5 g, fumarate = 0.725 g, fructose = 1 g, citric acid = 50 mg, zinc = 10 mg, coenzyme Q10 = 20 mg, selenium = 50 µg, Vit C = 90 mg, folic acid = 200 µg, Vit B12 = 1.5 µg daily for 3 months	Idiopathic oligoasthenozoospermia (Placebo group = 50, Treatment group = 50)	Increase in semen volume, progressive motility, and vitality in placebo group. Decrease in sperm DNA fragmentation index in treatment group.	[76]
5 mg folic acid, 30 mg zinc once daily for 6 months	1773 men planning to undergo infertility treatment with spouse	No improvement in S_mot_ and S_morph_	[77]

SC = sperm concentration, S_mot_ = sperm motility, and S_morph_ = sperm morphology, DFI = DNA fragmentation index.

## Abbreviations

AAS = anabolic–androgenic steroids; CoQ10 = coenzyme Q10; E2 = estradiol; ER = endoplasmic reticulum; DFI = DNA fragmentation index; ESHRE = European Society for Human Reproduction and Embryology; FDA = Food and Drug Administration; GSH = reduced glutathione; GP_X_ = glutathione peroxidase; GSSG = oxidized glutathione; ICSI = intracytoplasmic sperm injection; IVF = in vitro fertilization; keap1 = Kelch-like ECH associated protein 1; LPO = lipid peroxidation; MAPK = mitogen activating factor kinase; MetS = metabolic syndrome; NFkB = nuclear factor kappa B; NO = nitric oxide; Nrf2 = nuclear factor-erythroid factor-2-related factor-2; PG = prostaglandin; PUFA = polyunsaturated fatty acids; ORP = oxidation-reduction potential; OS = oxidative stress; RNS = reactive nitrogen species; ROS = reactive oxygen species; SOD = superoxide dismutase; UPR^ER^ = unfolded protein response of the endoplasmic reticulum.
